# The mediating role of dispositional resilience in the relationship between spirituality and life satisfaction of Filipino older adults

**DOI:** 10.3389/fpsyg.2025.1570762

**Published:** 2025-08-07

**Authors:** Stephanie Joy Adriano Gusilatar, John Lloyd Calungsod, Akira Garcia, Beatriz Santos, Wawie De Guzman Ruiz

**Affiliations:** Department of Psychology, College of Arts and Social Sciences, Central Luzon State University, Science City of Muñoz, Nueva Ecija, Philippines

**Keywords:** dispositional resilience, spirituality, Filipino, life satisfaction, mediation, Filipino older adults

## Abstract

The study examined the relationship between spirituality and life satisfaction of Filipino older adults as mediated by dispositional resilience. Guided by the Selection, Optimization, and Compensation (SOC) model, the study employed a quantitative correlational design and a sample of 211 elderly Filipinos from Nueva Ecija, Philippines. Prior studies had established a nonlinear relationship of spirituality and life satisfaction, in contrast, this study attempted to provide a more definitive path between spirituality and life satisfaction. Moreover, previous researchers uncovered mediating effects of dispositional resilience with other concepts, whereas this study aimed to identify its role when mediating the aforementioned variables. Consequently, the findings revealed that spirituality and life satisfaction are positively correlated to dispositional resilience, the direct relationship of spirituality to life satisfaction was not significant. However, the effects of spirituality to life satisfaction can be amplified through the mediating effect of dispositional resilience. This indicates that dispositional resilience plays a significant role in understanding the influence of spirituality to the life satisfaction of Filipino older adults.

## Introduction

Spirituality is a concept that is often associated with successful aging. It is evident, from an extensive amount of studies, that as people gradually age, their levels of spirituality simultaneously increases. According to (Soriano et al. 2022) in their study regarding spirituality, religiosity, and death anxiety; the degree of spirituality of Filipino older adults living within a community and in healthcare institutions are particularly high, especially those living in healthcare institutions. Moreover, recent studies also consistently show that people with higher levels of spirituality tend to have better mental health and show more effective coping mechanisms than individuals who have lower levels of spirituality. In line with this, the study of (Reis and Menezes 2017) also highlights the importance of spirituality not only to mental health but more importantly, to the physical health of people in old age, their overall risk for diseases, and their response to medical treatment.

Existing research indicates a complex, non-linear relationship between spirituality and life satisfaction (Okulicz-Kozaryn, 2010), influenced by factors such as physical health, mental health, aspirations, and perceptions of success (Bhullar, 2013). Additionally, dispositional resilience has been identified as a mediating factor in this relationship, particularly among individuals facing significant life challenges (Rahman et al., 2020). However, the specific mechanisms driving variability in life satisfaction among spiritual individuals remain unexplored. This study aims to address these gaps by examining the nuanced interactions between spirituality, dispositional resilience, and other factors influencing life satisfaction, considering cultural and contextual variations. Alternatively, dispositional resilience has been demonstrated to have a positive association with life satisfaction. For instance, (Cohn et al. 2009) found a positive correlation between dispositional resilience and life satisfaction. According to transactional stress theory, personality traits such as dispositional resilience play a crucial role in the appraisal process, which subsequently influences the relationship between stressors and stress responses. However, despite these findings, no study, to the best of our knowledge, has yet been conducted to explore the potential mediating role of dispositional resilience in the relationship between spirituality and life satisfaction.

Moreover, investigations also revealed that increased spirituality is connected with decreased feelings of despair amongst individuals in later life. Furthermore, it is also noteworthy to take into account that there is a positive relationship between spiritual health and the life satisfaction of elderly as reflected in the study of (Aslani et al. 2018).

On the other hand, recent studies regarding spirituality such as (Reis and Menezes, 2017) and Manning (2013), asserted that people in their old age utilizes spirituality as a tool which promotes their dispositional resilience in later life. Consequently, their investigations contended that dispositional resilience is rooted in the spirituality of people in old age and these individuals consider spirituality as strategies in dispositional resilience. Some investigations highlight the importance of spiritual engagement not only to the mental health but also to the physical health of people in old age, influencing their overall risk for diseases and determining their response to medical treatment. In fact, studies that focused specifically on spirituality have generally found strong associations with both mental and physical health (Ballew et al., 2012), further supporting the significant role of spiritual involvement in promoting wellbeing among the elderly.

The need for an investigation regarding the possible influence of spirituality to the life satisfaction of people in old age is an attempt to establish a more concrete connection between the two concepts. Moreover, older Filipino adults tend to have a strong sense of spirituality (Pausanos 2013), which made it a ground for the researchers to further explore other potential influences of spirituality to the life satisfaction of older adults as mediated by dispositional resilience. Although the present study incorporates dispositional resilience as a mediating variable, it is utilized to explain the mechanism behind the levels of spirituality and life satisfaction of Filipinos in old age. More importantly, the study will contribute to the further development of understanding toward spirituality that is independent from the concept of religiosity, given that researchers more often assess religiosity compared to spirituality (Kaplan 2023).

### Aging in the context of Filipino

The elderly population in the Philippines is steadily increasing, reaching 9.22 million in 2020 from 4.6 million in 2000 due to longer life expectancies. This growth presents challenges, including inadequate social pensions for 4.1 million indigent seniors and limited access to healthcare, worsened by high mortality from preventable diseases and a lack of geriatric wards in government hospitals (Giron and de la Vega, 2022). In addition, the Philippines has proactively developed a comprehensive national policy to address these challenges, with a focus on promoting the wellbeing and active participation of seniors, prioritizing their health, and fostering an age-friendly environment, thus underscoring the nation's commitment to inclusive policies.

The Philippines has grappled with addressing the concerns related to an aging population, particularly because nearly 5% of the country's current population falls into this disadvantaged age group (Badana and Andel, 2018; Salvador and Alqahtani, 2020). As for the findings, the collected life stories have revealed four distinct recurring themes: the prevalence of age discrimination, the distress caused by mistreatment of the elderly, the resilience exhibited in overcoming challenges, and the necessity of active community involvement to shape a future that offers elderly individuals the dignified life they deserve and helps them realize their potential, despite their limited time. This study underscores the importance of comprehending the experiences of elderly Filipinos as a practical means to evaluate the requirements of this demographic for a life marked by respect and honor. Ultimately, this qualitative research emphasizes the critical social responsibility of caring for and cherishing the elderly in our society (Salvador and Alqahtani, 2020).

Elderly individuals who enjoy good health, are single, belong to the middle or upper social classes, possess at least a college-level education, engage in regular leisure activities and exercise, maintain strong communication ties with close family and friends, and actively participate in religious activities are generally considered more likely to experience successful aging among Filipino elders. However, factors such as age, gender, occupation, residential location, ongoing education, communication methods with family and friends, religious affiliation, and commitment to lifelong learning were not found to be significantly correlated with successful aging in elderly individuals (Del Villar, 2015). Meanwhile, to achieve successful aging, high-power distance, collectivism, and subjectivity to nature are the three cultural orientations that were deep-rooted to a Filipino's heart and mind (Del Villar, 2015).

Several studies have indicated that subjective wellbeing remains stable or even increases well into old age. Middle-aged individuals appeared more susceptible to experiencing low subjective wellbeing and mental health issues. However, as individuals age, they are anticipated to encounter a buildup of adverse life circumstances, such as illness and disability, as well as significant life events like the loss of companionship. These conditions could potentially impact their subjective wellbeing. Supporting this notion, a longitudinal study demonstrated that subjective wellbeing tended to decrease only after reaching the age of 70.

Therefore, the challenge lies in comprehending the factors influencing the subjective wellbeing of the aging population. Also, this study has revealed various perceptions of Filipino elderly individuals toward aging. These include viewing it as a responsibility, a maturation period, a promising and positive experience, a process that leads to increased autonomy, a phase for enhanced productivity, an apprehensive process, a stage marked by physical decline, and a phase of physiological changes (Valdez et al., 2013).

### Spirituality

This study conceptualizes spirituality as defined by (Koenig et al. 2001), spirituality entails an individual's pursuit of profound insights into life's fundamental inquiries concerning purpose, significance, and connection to the divine or transcendent. Additionally, Koenig (2008) also employed the term “spirituality” to characterize the endeavors of those who wholeheartedly commit themselves to religious service or embody the principles of their faith traditions. The conceptualization of spirituality does not limit the current study to conform to a singular spiritual affiliation. Thus, it is necessary to recognize all levels of spirituality of the participants regardless of their religious and spiritual background as long as they are living within the community.

### Spirituality and dispositional resilience

Dispositional Resilience is frequently viewed as an innate attribute or individual quality that empowers individuals to confront difficulties and prosper despite them, typically observed in those with a robust disposition (Richardson 2002; Sinclair and Oliver, 2003; Sagone and Caroli, 2014). Meanwhile, dispositional resilience is conceptualized as “a personality trait with three aspects: commitment, control, and challenge (Bartone et al., 2008; Kobasa, 1979)”.

The concept of dispositional resilience is emphasized as an inherent adaptive trait possessed by individuals, empowering them to flourish despite encountering traumatic and highly stressful situations.

One of its core components, commitment, refers to a person's tendency to fully engage in life's activities and maintain meaningful involvement in daily tasks and relationships. Kobasa (1979) described commitment as a genuine interest and curiosity about the world—including people, events, and experiences—along with a sense of personal competence and belongingness to a community or organization. Control and challenge is another aspect of dispositional resilience (Bartone et al., 2008; Kobasa, 1979). In regards to this, (Choudhary et al. 2022) discussed that spirituality as an intervention and a self-control technique for coping with stress and challenges in later life has grown. In the context of elderly individuals residing in nursing homes, the challenges are amplified. The discourse highlights spirituality as a potent tool for alleviating loneliness, promoting good mental health, and overcoming the myriad obstacles that individuals encounter in the final phase of their lives.

In various studies, such as Manning (2013), spirituality is considered as a promoter of healthy aging in older adults and those people in their 80's and older use spirituality as a tool that promotes and maintains their dispositional resilience in later life. Moreover, (Reis and Menezes2017) found out in their study with “long-living” older adults that religiosity and spirituality are considered as strategies for dispositional resilience. Manning (2013), asserted that dispositional resilience is firmly rooted with the spirituality of older women which is evident from the recurring themes in the analysis of the interviews with the participants that stressed the role of spirituality as a mechanism in dealing with their adversities and opportunities in later life. The study adapted Atchley's (2009) concept defines spirituality as a “domain of human experience encompassing a heightened awareness of the present moment, transcending the personal self, and/or a sense of connection with all life, the Universe, or a Supreme Being.”

(Reis and Menezes2017) found out in their study, that spirituality and religiosity are significant dispositional resilience strategies for older people, since it is spirituality and religiosity that helps them attain wellbeing, cope with late-life challenges, recovery/sustaining good health, and more importantly, experiencing successful aging. Additionally, It is also important to note that spirituality does not only correlate with dispositional resilience, but it also functions as a vital coping resource in the face of adversity and life challenges (Tamparon Aragon and Cardoza, 2023).

Meanwhile, according to (Moreira et al. 2020) the elderly population, particularly those aged 80 and above, perceive religiosity and spirituality as sources of inner fortitude for confronting life's challenges and imbuing life with significance. Individuals in this age group possess heightened requirements for religiosity and spirituality, esteeming religious convictions and rituals as pivotal, employing them as sources of dispositional resilience in coping with life's adversities, and drawing upon them to find purpose in their existence. Further, Nunes (2016) concluded that the presence of spirituality as a valuable source of support, leading to increased dispositional resilience and the capacity to conquer challenges in various life situations. In addition, humor and spirituality are two inner strengths that can help older adults to have resiliency. Finding ways to infuse humor into everyday life and activities, as well as seeking spiritual support, can promote dispositional resilience in older adults living in the community (Kenaley et al., 2019).

### Dispositional resilience

In the study of (Hayat et al. 2016) regarding the relationships between wisdom, resilience, and life satisfaction of both elderly adults that live with their families and old-age home; the results of their investigation revealed that resilience has a significant and positive relationship with life satisfaction. It is also noteworthy that dispositional resilience mediated the relationship between wisdom and life satisfaction. Additionally, the investigation pointed out that the level of dispositional resilience of elderly adults living with their families are more resilient, have more wisdom, and are more satisfied in life in comparison to elderly adults living in Old-Age homes. Also, (Ye and Zhang2021) meta-analysis study showed that there is a significant positive relationship between dispositional resilience, wellbeing, satisfaction with life, and positive emotions among elderly.

On the other hand, Shimono (2021) presented various studies discussing the relationship between dispositional resilience and life satisfaction such as the mediation and moderation of dispositional resilience between perceived stress and life satisfaction (Rossi et al., 2007). Dispositional resilience was favorably connected with positive affect and negatively correlated with negative affect, which are two of the three elements of subjective wellbeing and stand in for life satisfaction. In addition to the five-factor personality traits, dispositional resilience predicted both positive and negative effects (Kardum et al., 2012). In a study of widows, dispositional resilience was found to be positively related to life satisfaction; additionally, it has both mediated and moderated the link between perceived stress and life happiness (Rossi et al., 2007).

### Spirituality and life satisfaction

Spirituality and life satisfaction are integral psychological aspects that significantly influence the wellbeing of elderly individuals. Recent studies have emphasized the pivotal role of spirituality as a predictor of late-life functioning and its potential value in addressing the unique challenges that come with aging. Spirituality can provide a valuable framework for addressing existential questions related to the later stages of life. As people age and confront issues such as mortality and the search for meaning, their spiritual beliefs can offer solace and guidance contributing to a sense of inner peace and overall life satisfaction. Diener (1984) quoted life satisfaction as “an overall assessment of attitudes and feelings about one's life at a particular point in time spanning from negative to positive” (Beutell 2006; Diener, 1984).

The study conducted by (Cowlishaw et al. 2012) yielded a noteworthy finding: spirituality demonstrated a positive and enduring connection with heightened life satisfaction among the elderly. In other words, as spirituality increased, so did the sense of meaningfulness in life, which in turn contributed to elevated levels of life satisfaction over an extended period.

(Rezaieshahsavarloo et al. 2016), further corroborated these findings. Their research underscored the significance of spiritual wellbeing and religious attitudes in enhancing the life satisfaction of the elderly population. This underscores the quantitative importance of spirituality as a predictor of life satisfaction among older adults.

Moreover, (Rivera et al. 2019) supported the claim of Koenig (2012) and (Maheshwari and Singh2009) that it has a significant positive relationship between the spiritual attitudes, and life satisfaction. Also, (Rivera et al. 2019) provided additional support in the study made by Koenig (2012) and (Maheshwari and Singh2009) regarding the substantial and positive correlation between religious and spiritual attitudes and life satisfaction among the elderly. Their study delved into the idea that increased spirituality is linked to decreased feelings of despair among individuals in their later years. Additionally, spiritual wellbeing was significantly related to life satisfaction and hope (Özdemir et al., 2023).

On the other hand, according to Koenig (2012), religious and spiritual beliefs and practices are frequently utilized by both medical and psychiatric patients as coping mechanisms in the face of illness and other stressors that occur during the various stages of life, including old age. An extensive body of research consistently demonstrates that individuals with higher levels of spirituality tend to EXPERIENCE better mental health and exhibit more effective adaptation when confronted with health-related challenges compared to those with lower levels of religiosity or spirituality.

Meanwhile, (Aslani et al. 2018) studied the relationship between spiritual health and life satisfaction in the elderly people. Results revealed that there was a positive, significant relationship between the variables. Also, the majority of elderly individuals reported a low level of spiritual health, yet surprisingly, their level of satisfaction remained high. On the contrary, having a negative view of God significantly has lower life satisfaction, leading to adverse effects on the mental and physical wellbeing of religious individuals (Lãng, 2013).

Similarly, a number of researches have demonstrated a favorable association between spirituality, religion and life satisfaction in the cognitive component of wellbeing (Yoon and Lee, 2004). To explain these results, it has been proposed that those who feel more connected to and directed by a higher power, i.e., people who engage in religious and spiritual activities, have a more positive view of their life (Vishkin et al., 2019; Ramsay et al., 2019). The sensation of being connected to a greater power, people, and life in general is an effective approach to preserve a positive perspective on one's life, despite all the probable unpleasant situations that one may face. Furthermore, spiritual participation may help people's lives by boosting both internal (e.g., a sense of self-worth) and social (e.g., a sense of belonging to a community) resources (Lim and Putnam, 2010).

In essence, these additional studies, along with the work of Koenig et al. underscore the quantitatively robust connection between spirituality and life satisfaction among the elderly. This relationship not only affects mental wellbeing but also has far-reaching implications for physical health, making it a critical area of exploration in the field of gerontology and healthcare.

### Dispositional resilience as a mediator

Several studies highlight the critical role of dispositional resilience as a mediating factor in the relationship between early life adversity and mental health outcomes in later life. (Li et al. 2023) found that dispositional resilience helps explain how childhood trauma contributes to depressive symptoms among older adults, suggesting that those with higher trauma exposure may have weakened coping mechanisms, leading to increased vulnerability to emotional distress. Drawing from the Stress Process Theory, this finding supports the idea that resilience buffers the impact of early trauma on mental health.

Similarly, (Platania et al. 2022) emphasized the importance of dispositional resilience in mitigating the effects of psychological stressors, such as depression, stress, and anxiety, particularly in relation to professional quality of life. Their findings indicate that resilience plays a protective role by enhancing positive psychological outcomes like compassion satisfaction.

In a related study, (Vieira et al. 2020) also identified dispositional resilience as a key factor linking childhood trauma to mood disorders in young adults. Their research suggested that resilience helps reduce the negative impact of trauma on emotional wellbeing, even though some direct effects of trauma may persist.

Moreover, Fangauf (2014) found that dispositional resilience mediates the relationship between spirituality and both depression and life satisfaction. The study underscores that spirituality, together with resilience, can significantly contribute to improved mental wellbeing.

These studies underscore the significance of dispositional resilience as a key psychological factor that helps buffer the negative effects of trauma and stress throughout life. Highlighting the potential of dispositional resilience to serve as a crucial link through which spirituality contributes to enhanced life satisfaction in later adulthood.

### The current study

The study explored the potential role of dispositional resilience as a mediator in the relationship between spirituality and life satisfaction among elderly people in the Filipino community, addressing a gap in the literature and providing valuable insights to improve the wellbeing of this population. This research sought to uncover the dynamics in the relationship between spirituality and life satisfaction and explore any mediating roles that dispositional resilience may have played, thereby providing practical insights for enhancing the quality of life for older adults and inspiring future research into the dynamics of aging and wellbeing within the community. This study's significance lay in its contribution to understanding the wellbeing of Filipino older adults by examining the interplay between spirituality, dispositional resilience, and life satisfaction. It had practical implications for interventions and support services, addressed cultural relevance, revealed mediating mechanisms, informed policies, and added to academic knowledge in gerontology, psychology, and spirituality.

The related literature from this study fundamentally acknowledged the spirituality and life satisfaction of Filipino older individuals in society. It particularly detailed the importance of these factors in achieving the aims of the elderly for their living. The revealed findings provided significant support for the main objectives of the current study, which were to discover the: (1) relationship between spirituality and dispositional resilience; (2) relationship between dispositional resilience and life satisfaction; (3) relationship between spirituality and life satisfaction; (4) mediating role of dispositional resilience in the relationship between spirituality and life satisfaction.

### Theoretical framework

Drawn by the Selection, Optimization, and Compensation (SOC) model developed by (Baltes and Baltes1990), our study emphasized the significant role of dispositional resilience as a mediator and explained the mechanism behind the relationship between spirituality and life satisfaction. According to this theory, developed by (Baltes and Baltes1990), selection involves choosing and prioritizing goals in response to limited resources, like reduced physical or cognitive abilities. There are two types: “loss-based selection,” where goals are abandoned involuntarily, and “elective selection,” where goals are prioritized based on personal preferences. Optimization entails investing resources to achieve goals, such as training or acquiring knowledge, while compensation involves using alternative means or external resources. Together, these strategies aim to maximize gains and minimize losses associated with aging, promoting successful development and aging.

In the context of our findings, dispositional resilience was established as a critical mediator that links spirituality and life satisfaction, and its mediating role can be best understood through the lens of the SOC model. Our results suggest that elderly individuals strategically engage in SOC-based behaviors to adapt to aging, especially through their spiritual orientations and resilient dispositions. The SOC model allows for a theoretically grounded interpretation of how Filipino older adults manage declining resources, such as physical strength or social roles, while maintaining life satisfaction through inner capacities like dispositional resilience and spirituality. Elderly people chose to engage in spiritual practices or beliefs that were meaningful to them, aligning with the concept that selection involved choosing activities based on values and preferences, such as spirituality offering solace, meaning, and connection, particularly in later life. This elective selection reflects a conscious decision to allocate personal and emotional resources toward enhancing their spirituality. In our study, this behavior was evident among the majority of respondents who identified as Christian, indicating a deliberate prioritization of spiritual goals driven by cultural and personal values.

Furthermore, optimization, in relation to dispositional resilience, fortified the bond between spirituality and life satisfaction by actively utilizing resources to enhance wellbeing. Our study suggested that dispositional resilience played a significant role in this process, as individuals used their spiritual beliefs to cope with adversities and foster dispositional resilience. Resilient individuals turned to practices such as prayer or meditation for strength and comfort during difficult situations. These practices serve as optimization strategies, enhancing their capacity to meet challenges by strengthening psychological resources.

Lastly, in life satisfaction, compensation was employed when facing limitations or losses. In this framework, compensation involved finding alternative ways to maintain or regain functioning despite challenges. Dispositional resilience served as a compensatory factor, allowing people to rely on their spirituality to cope with stress and maintain overall life satisfaction. For example, when dealing with health challenges or the loss of loved ones, resilient people relied on their spiritual beliefs to provide dispositional resilience and inner strength. Here, dispositional resilience acted as a functional buffer—compensating for external losses with internal adaptability and meaning-making processes.

In adulthood, there are two theoretically plausible hypotheses regarding how the Selection, Optimization, and Compensation (SOC) model develops over time. The first hypothesis proposes that as people become older, they get more adept at using the SOC model as a result of their life experiences. The second hypothesis posits that the biological and physical limitations of aging cause a decrease in resources, potentially limiting the adoption of the SOC model. As a result, in old age, SOC-related activities may decline. This suggests a developmental trajectory where the utilization of SOC strategies initially rises with age before declining (Zajac-Lamparska, 2021; Freund and Baltes, 2002; Baltes and Baltes, 1990). The results of this study align with the former trajectory, highlighting that Filipino elderly individuals leverage SOC strategies—particularly through dispositional resilience—to mediate the impact of spirituality on life satisfaction. This suggests that dispositional resilience not only fits within the SOC framework but also operates as a central mechanism through which selection, optimization, and compensation are carried out in daily life.

The Selection, Optimization, and Compensation (SOC) model provided a framework for understanding how individuals adapted to aging. By selectively choosing goals, actively optimizing resources, and employing compensatory strategies, individuals effectively navigated challenges and promoted life satisfaction. Dispositional resilience emerged as a key factor, enabling individuals to leverage their strengths and adaptability to enhance optimization efforts and cope with adversities, ultimately contributing to satisfaction in later life.

Dispositional resilience as a mediator in this study reflects the three-dimensional mechanism of the SOC model. The integration of this theoretical model clarified how spirituality influences life satisfaction primarily through resilience-based selection, optimization, and compensation strategies, thereby providing a holistic explanation of psychological wellbeing among older Filipino adults.

### Conceptual framework

Prior research has consistently shown a positive association between spirituality and dispositional resilience, as well as between dispositional resilience and life satisfaction. Building on this existing literature, our study aimed to delve deeper into the intricate dynamics among these variables within the context of elderly individuals in the Philippines, shedding light on the mechanisms that contributed to positive aging.

**Figure 1:**
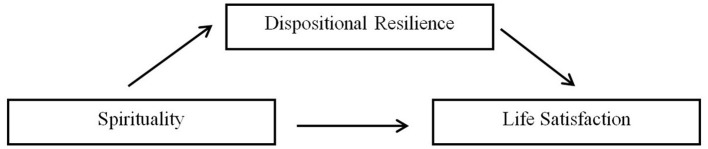
Conceptual model of variable interactions.

In this investigation, spirituality was posited as the independent variable, with life satisfaction serving as the dependent. At the heart of this study lay the mediating variable, dispositional resilience, which was hypothesized to play a pivotal role in elucidating the link between spirituality and life satisfaction in the elderly population. Hence, establishing correlations among spirituality, dispositional resilience, and life satisfaction served to unveil the interconnectedness of these factors and their collective impact on the life satisfaction of Filipino old age individuals (Figure 1).

## Materials and methods

### Research design

This study employed a quantitative correlational design in order to understand the underlying mechanisms behind spirituality, dispositional resilience, and life satisfaction of elderly Filipino individuals.

#### Participants

This study used purposive non-probability sampling to select 211 elderly Filipinos (53% female, 47% male) aged 60–80 (M = 71.36) from Nueva Ecija, Philippines. Participants met two criteria: being within the age range and able to read, write, and understand Filipino. Of the respondents, 69.2% were married, 19.9% separated, and 10.9% widowed; 19.4% were college graduates, 42.7% had secondary education, and 37.9% reached elementary level. The sample included individuals living independently, retired, or residing with family.

### Instrumentations

At the beginning of the questionnaire, elderly Filipino participants were provided with informed consent and they responded to questions pertaining to their age, sex, religion, civil status, educational attainment, living arrangements, and employment status, as well as their religious affiliation.

#### Spirituality

Filipino Spirituality Scale (Yabut 2017). This scale consists of 46-items and has a strong reliability with a Cronbach Alpha score of 0.978. Likert scale was used scaling from 1 (Hinding-hindi totoo tungkol sakin) to 5 (Totoong-totoo tungkol sakin).

#### Life satisfaction

Satisfaction With Life Scale (Diener et al., 1985) is a 5-item questionnaire with a strong internal consistency, with a strong alpha coefficient of 0.87, and it also exhibits outstanding test-retest reliability, showing a correlation of 0.82 over a 2-month interval. Likert Scale was used in this scale from 1 (strongly disagree) to 7 (strongly agree).

#### Dispositional resilience

Dispositional Resilience Scale (Bartone 2013) version 3.0 is a questionnaire comprising 15 items that displayed a strong internal consistency of 0.83. Items in this scale were rated on a scale ranging from 0 (not at all true) to 3 (completely true). Items marked with an asterisk (^*^) had reversed their scores.

### Data gathering procedures

Information was gathered from a sample of 211 old age Filipino individuals residing in Nueva Ecija, Philippines. To ensure a smooth data collection process, researchers conducted one-on-one sessions with each participant, providing clear verbal and written instructions alongside the assessment tools. Additionally, the researchers collaborated with Barangay Officials to facilitate easy access to potential participants, streamlining the recruitment process.

Upon concluding the data collection phase, participants were offered a debriefing session, during which any inquiries or concerns they may have had were addressed, ensuring their complete understanding and comfort with their involvement in the study.

### Data analysis

The data collected was subjected to a rigorous analysis process aimed at extracting meaningful and descriptive interpretations from the outcomes of the study. This study encompassed three key variables: spirituality, dispositional resilience, and life satisfaction. These variables underwent thorough statistical analysis using IBM SPSS Statistics 20, JASP software, and Jamovi Statistical Software, robust tools for such assessments. The primary objective was to ascertain whether statistically significant associations existed among these variables, particularly exploring the interplay between spirituality, dispositional resilience, and life satisfaction.

For the data analysis process, the dataset was carefully examined in alignment with the study's objectives. This in-depth scrutiny facilitated a comprehensive evaluation of the levels of spirituality, dispositional resilience, and life satisfaction prevalent among the elderly Filipino individuals. For this purpose, Descriptive Statistics were applied. For the mediating role of dispositional resilience, a series of mediation analysis was employed in the study by utilizing the Hayes Process Macro. Subsequently, the research delved into the correlation between these variables. To explore these relationships, the researchers employed the Pearson Product Moment Correlation Coefficient, a statistical technique that enabled the investigation of potential connections and dependencies among spirituality, dispositional resilience, and life satisfaction in the context of Filipino old age.

### Ethical considerations

In this research study, strict ethical measures were taken to safeguard participants' rights, wellbeing, and privacy. These ethical considerations encompassed informed consent, where participants received comprehensive information about the study's purpose, procedures, potential risks, and benefits, with the freedom to ask questions and withdraw from the study at any time. Confidentiality measures included anonymizing data to protect participants' identities, ensuring that they could not be identified from the research results or publications. Moreover, data security protocols were in place, both electronically (through encryption and password protection) and physically.

## Results

The current study aims to investigate the mediating role of Dispositional Resilience (DR) in the relationship between Filipino Spirituality (FS) and Life Satisfaction (LS). The mediating effect(s) of Dispositional resilience is examined using multiple regression and mediation analysis.

Data were gathered from a sample of old-age Filipino individuals (*N* = 211) with an age range of 60 to 80 years old (*m* = 71.35, *SD* = 6.22). The participant's level of life satisfaction reported an average of 24.03 (*SD* = 6.10), which indicates that the respondents are only slightly satisfied with their life. Moreover, assessment suggests average levels of dispositional resilience (*m* = 28.79, *SD* = 4.83), while ratings of Filipino spirituality ranged from 2.59 to 5 (*m* = 4.77, *SD* = 6.22) indicating high levels of spirituality. Refer to Table 1.

**Table 1:** Descriptive statistic.

**Variable**	**Mean**	***SD***	**1**	**2**	**3**	**4**
Age	71.35	6.22	-			
Life satisfaction	24.03	6.10	-0.09	-		
Dispositional resilience	28.79	4.83	-0.17^*^	0.46^***^	-	
Filipino spirituality	4.77	0.39	-0.08	0.189^**^	0.56^***^	-

^*^p < 0.05.

^**^p < 0.01.

^***^p < 0.001.

To investigate the relationship of the variables, preliminary correlation analysis was conducted using Pearson's correlation, initial analysis revealed a positive relationship between variables. The results suggest that there is a significant positive correlation between spirituality and life satisfaction *r* = 0.18, at *p* < 0.01. Moreover, it is also revealed that there is a strong significant correlation between spirituality and dispositional resilience reporting an *r* = 0.56 at *p* < 0.001. Findings also revealed that there is a significant correlation between dispositional resilience and life satisfaction with *r* = 0.46 at *p* < 0.001, strong correlation between the three variables was established.

The mediating effect of dispositional resilience was examined through a series of mediation analysis using Hayes Process Macro Model 4. To probe more deeply into understanding the mediational role of dispositional resilience in the relationship between spirituality and life satisfaction of old-age Filipino individuals, it is important to investigate and describe the pathways in the proposed mediation model. Preliminary correlation analysis of the relationship between spirituality and dispositional resilience suggests that there is a significant positive correlation between the two variables. The mediation model recognizes this relationship as the first pathway (path a) and the result of the mediation analysis is in line with the initial correlation reporting β = 0.56, 95% *CI* = 0.45–0.67 (*p* < 0.001) further establishing strong positive correlation of variables. Subsequently, the relationship between dispositional resilience and life satisfaction (path b) rendered (β = 0.51, 95% *CI* = 0.37–0.66, *p* < 0.001) indicating significant positive correlation. On the other hand, the mediation analysis revealed that there is no existing relationship between spirituality and life satisfaction within the presence of the mediator as indicated by the resulting path coefficients (β = −0.10, 95% *CI* = –0.24–0.04, *p* = 0.159). Refer to Table 2.

**Table 2:** Mediational analysis pathway coefficients.

**Path**			**95% CI**						
			**Label**	**Estimate**	***SE***	**Lower**	**Upper**	***z***	***p***
Spirituality	→	Dispositional resilience	a	0.56	0.05	0.45	0.67	9.90	< 0.001
Dispositional resilience	→	Life satisfaction	b	0.51	0.07	0.37	0.66	7.06	< 0.001
Spirituality	→	Life satisfaction	c	−0.10	0.07	−0.24	0.04	−1.41	0.159

Mediation analysis revealed that the direct effect of spirituality on life satisfaction in the presence of the dispositional resilience only accounts for 26.2% effect in the mediation model and suggested no significance inferring from the resulting path coefficient (β = −0.10, 95% *CI* = −0.24–0.04, *p* = 0.159), this result is in contrast with the initial correlation conducted. On the other hand, the indirect effect or the effect of spirituality to life satisfaction through the mediation of dispositional resilience yielded significant findings. Accounting for 73.8% of the effect in the model, dispositional resilience mediates the relationship between spirituality and life satisfaction reporting β = 0.29, 95% *CI* = 0.19–0.39, *p* < 0.001, significance of this mediation was examined through bootstrap method with 5,000 resample; provided that the direct effect is insignificant it is inferred that dispositional resilience fully mediates the relationship between spirituality and life satisfaction. Consequently, total effects pathway (β = 0.18, 95% *CI* = 0.05–0.32, *p* = 0.005) further corroborates the finding that the initial relationship of spirituality with life satisfaction can be amplified through mediation of dispositional resilience (Table 3). These results suggest that dispositional resilience as a trait of Filipino old-age individuals significantly influences their life satisfaction exponentially, more than what their spirituality can predict.

**Table 3:** Mediational role of dispositional resilience in the relationship between Filipino spirituality and life satisfaction.

**Mediation estimates**		**β**	***SE***	**95% Confidence interval**		***Z***	***p***	**% Mediation**
**Effect**	**Path**			**Lower**	**Upper**			
Indirect	a × b	0.29	0.05	0.19	0.39	5.75	< 0.001	73.8
Direct	c'	−0.104	0.0736	−0.2478	0.04	−1.41	0.159	26.2
Total	c + a × b	0.18	0.06	0.05	0.32	2.79	0.005	100.0

## Discussion

The main objective of this study was to examine the mediating role of dispositional resilience in the relationship between spirituality and life satisfaction of Filipino older adults. Secondly, this study investigated the relationship of spirituality and dispositional resilience and determined the relationship of dispositional resilience and life satisfaction. The findings are examined within the context of the research's objectives, drawing comparisons with the literature reviewed. This study provided comprehensive evidence supporting all proposed hypotheses, with interpretations framed under the overarching concept of spirituality in the context of Filipino culture.

The discussion is structured around four key points: (a) interpretation of current findings in light of prior literature, (b) the significance of the mediating effect, (c) cultural implications within the Filipino context, and (d) recommendations for further inquiry.

### Advancing the field: interpreting findings through a contemporary lens

**Figure 2:**
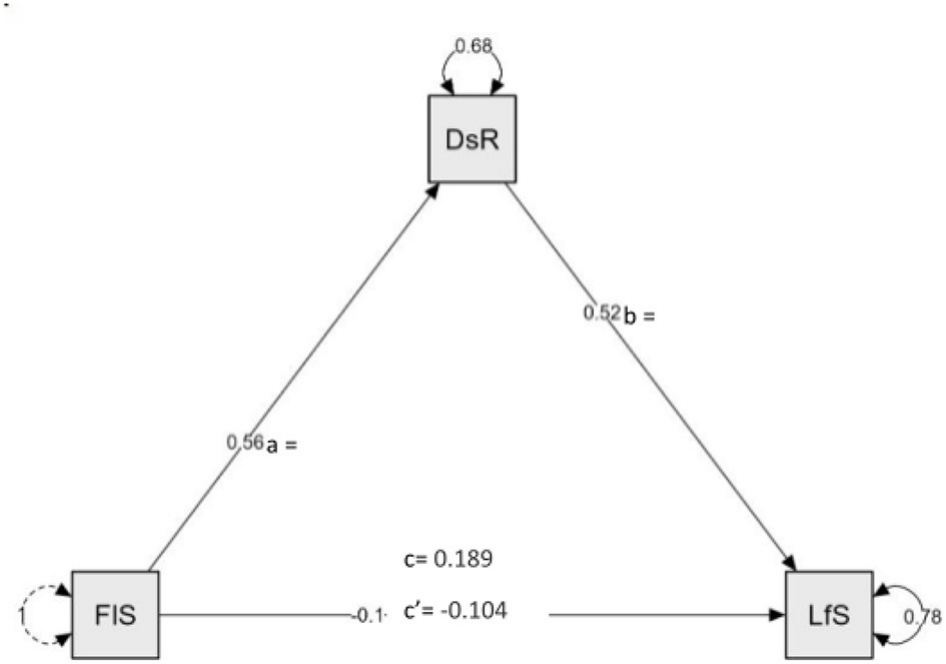
The mediating role of dispositional resilience in the relationship between Filipino spirituality and life satisfaction.

This study makes a distinct contribution by emphasizing the mediation effect of dispositional resilience in spirituality–life satisfaction linkage. While previous literature has often cited a direct connection between spirituality and life satisfaction, our data challenge this assumption within the Filipino context. The findings demonstrated that although spirituality is positively associated with dispositional resilience (path A) and dispositional resilience is positively associated with life satisfaction (path B), the direct relationship between spirituality and life satisfaction (path C) was not statistically significant (Figure 2). This nuanced conclusion refines existing understandings and supports the proposition that spirituality's role in wellbeing is more complex than previously reported.

Consistent with (Schwalm et al. 2021), this study confirms the role of spirituality in enhancing dispositional resilience through fostering values, meaning, and coping mechanisms. This supports the notion that spirituality influences internal strengths rather than life satisfaction directly. Similarly, the finding that dispositional resilience correlates with life satisfaction echoes results by Kalonia et al. (2022) and (Shi et al. 2015), reaffirming its vital role in navigating adversities such as aging, physical decline, and emotional stress.

However, the absence of a significant direct relationship between spirituality and life satisfaction contributes to a growing body of culturally grounded findings. For instance, Okulicz-Kozaryn (2010), Bhullar (2013), and (Mancuso and Lorona2023) uncovered that spirituality may not necessarily predict higher life satisfaction, especially when standards of satisfaction or socio-economic stressors are considered. This supports the assertion that individuals with high spirituality might report either very high or very low life satisfaction based on other contextual influences.

### Theoretical and practical implications

From a theoretical standpoint, the full mediation identified advances the applicability of the Selection, Optimization, and Compensation (SOC) model (Baltes and Baltes, 1990), wherein individuals optimize personal resources and select spirituality as a strategic response to life challenges. Dispositional resilience emerges as a core mechanism that operationalizes spirituality into psychological wellbeing. It reflects the “control” element within the SOC model—enabling older adults to regulate emotions and maintain a sense of purpose despite physical and social constraints.

Practically, this finding underscores the need to tailor wellbeing interventions for elderly Filipinos by not only encouraging spiritual practices but also developing resilience-oriented programs. Given the 73.8% mediation effect identified, nearly three-quarters of spirituality's influence on life satisfaction is channeled through dispositional resilience. This highlights the importance of resilience-building as a target for psychological services and community-based interventions in aging populations.

### Cultural implications in the Filipino context

This study situates its findings within the unique cultural narrative of the Filipino people, where spirituality is deeply embedded in daily life. Yet, despite this deep spiritual grounding, spirituality alone was not a significant predictor of life satisfaction—without the influence of dispositional resilience. This contradicts findings from Western contexts, where spirituality often has a more direct role in predicting life satisfaction. As noted by (Saroglou and Cohen2013) and (Sabatier et al. 2011), cultural norms and values significantly modulate spiritual experiences and their psychological effects.

### Limitations and directions for future research

While this study provides critical insights, it is not without limitations. First, the use of self-report measures may introduce biases such as social desirability or subjective misinterpretations of spirituality and life satisfaction. Second, the cross-sectional nature of the study restricts causal inferences. Third, while the sample captures a Filipino context, it may not fully represent the wide diversity of experiences among elderly populations across rural and urban settings or socio-economic strata.

Future research should consider longitudinal designs to establish causal links and explore potential moderators such as socio-economic status, health conditions, and the role of family and community support systems. Additionally, qualitative methods could deepen understanding of how elderly Filipinos define and operationalize both spirituality and resilience. Comparative studies across cultures would also be valuable to assess whether the mediating role of dispositional resilience holds across different belief systems and societal structures.

## Data Availability

The raw data supporting the conclusions of this article will be made available by the authors, without undue reservation.
